# Content analysis of school websites: policies and programs to support healthy eating and the environment

**DOI:** 10.1093/her/cyab040

**Published:** 2021-12-15

**Authors:** Neha K Lalchandani, Shona Crabb, Caroline Miller, Clare Hume

**Affiliations:** School of Public Health, The University of Adelaide, Level 4, 50 Rundle Mall Plaza, Adelaide, SA 5000, Australia; School of Public Health, The University of Adelaide, Level 4, 50 Rundle Mall Plaza, Adelaide, SA 5000, Australia; School of Public Health, The University of Adelaide, Level 4, 50 Rundle Mall Plaza, Adelaide, SA 5000, Australia; Health Policy Centre, South Australian Health and Medical Research Institute (SAHMRI), North Terrace, Adelaide, SA 5000, Australia; School of Public Health, The University of Adelaide, Level 4, 50 Rundle Mall Plaza, Adelaide, SA 5000, Australia

## Abstract

Preschools and primary schools are important settings for the development of healthy eating habits and awareness of environmentally friendly practices. This study explored South Australian government schools’ policies and programs in relation to healthy eating and environmentally friendly aspects of food choice (such as packaging), and whether any schools approached these issues in combination. Websites of 18 government preschools and primary schools in the Greater Adelaide region, stratified by low, medium and high socioeconomic status were reviewed for publicly available policies and other relevant content. A content analysis was undertaken, with policies and programs analysed deductively and thematically. Healthy eating (*n* = 8) and environment (*n* = 3) related policies were found on preschool websites only. The main themes observed across the three categories of interest (healthy eating, environmentally friendly practices and low-waste healthy foods) included the presence/absence of formal policy, promotional strategies and implementation. Expectations of children bringing healthy ‘nude’ foods that were environmentally friendly were mentioned informally on the websites but were not part of policy documents. Policies and programs around healthy eating and environmentally friendly practices (in combination) were lacking. There is scope to address this gap to improve health and sustainable outcomes within the school environment context.

## Introduction

The school environment has been recognized as an important setting for influencing eating behaviours and nutrition-related health outcomes of children and adolescents [1, 2]. Research investigating school food environments has found that policies and programs can have considerable impact on dietary patterns of school children [3]. The Australian school food environment is significantly reliant on food brought from home in the form of packed lunchboxes. This model is also found in Canada [4], unlike other school food models prevalent in the United States and United Kingdom [5], where paid school meal provision or government subsidized meals are often the norm, or in Sweden and Finland where free school lunches are provided [6]. Moreover, one-third of children’s daily energy intake occurs in the school setting [7] where they spend an average of 6 h per day and consume a packed lunch including a snack(s), for 5 days a week for a significant portion of the year. As a result, school food environments are an important setting for shaping children’s dietary habits [8].

Over a decade ago, several studies were conducted assessing the school food environment and eating patterns of Australian children, concluding that there was a lack of nutrition policy in schools that supported and promoted healthy eating among school children [9–11]. Given the availability of well-established evidence confirming the fundamental role of school food provision and policies in influencing children’s dietary behaviours [12, 13], improvements in lunchbox contents may be driven and sustained by school policies and programs [14]. Further, systematic reviews have shown that multi-component and multi-level interventions encompassing diet and physical activity, and involving parental engagement, along with environmental and policy components (such as healthy food policies that continually support behaviour change) were found to be beneficial in the context of obesity prevention in early care and education settings [15] and schools [16]. Thus, there is merit in exploring policies and programs around nutrition and health within the school food environment context.

Environmental concerns and their influence on dietary choices are of great interest given how overlaps have been identified and studied between health promotion and eco-friendly behaviours [17, 18]. Links between planetary health and dietary health, particularly those attributed to consumption patterns, have shaped recent public health agendas [19] and can be informed by the examination of the amount of food packaging and food waste generated in schools [20]. The concept of ‘litterless lunches’ has been translated to ‘Nude Food Day’ (for example) in Australia [21], an initiative strongly supported by Nutrition Australia [22] (an education and advisory service), where children’s lunchboxes are encouraged to be nutritious (not high in energy, saturated fat, added sugars and/or sodium or discretionary choices) [23] and devoid of packaging waste.

Evidence suggests that young children are able to learn and act on environmental issues through the guidance and influence of teachers [24, 25] and parents or guardians [25]. Elliott et al. [26] further affirmed that ‘Education for Sustainability’ approaches and pedagogies in early childhood communities will yield environmentally favourable outcomes. However, it is worth noting that although sustainability pedagogies do exist, they seem to be more ad hoc at a local level than systemic at a state or federal level in Australia [26]. Hence, it is worth exploring to what extent schools have policies and programs focusing on environmental attention and food consumption.

In this context, ‘policies’ refer to clearly defined and consistently informed requirements around nutrition and healthy eating for children, in line with the Australian Dietary Guidelines [23], National Quality Framework [27], the local guidelines such as the Right Bite strategy [28], and the Department for Education guidelines [29]. The Right Bite Healthy Food and Drink Supply Strategy is a guide developed to support South Australian preschools and school to not only supply healthy food and beverages, but also enable children to make better consumption choices [28]. A traffic light spectrum is used to categorize food and beverages into ‘Green category—choose plenty’, ‘Amber category—select carefully’ and ‘Red category—occasionally’.

While the causes of overweight and obesity conditions may be multi-factorial, environmental (public health strategies) and behavioural changes (individual health choices and actions) are important for the management and prevention of childhood obesity [30–32]. Exploring the avenue of food and packaging waste reduction could be part of an inter-sectoral approach to improve dietary habits of school children—a strategy that can potentially be complementary and synergistic [33]. Therefore, with a setting-based approach also in consideration, this study aimed to conduct a content analysis of the websites of preschools and primary schools to explore publicly available policies and programs around health and sustainability. This study sought to answer the research question: What is currently included in the policies and programs related to healthy eating and environmentally friendly practices (respectively or combined) in pre- and primary schools?

## Method

### Study design

A content analysis approach was used to identify policies and programs in relation to healthy eating, environmentally friendly practices and any combination of the two (what we will refer to hereafter as low-waste healthy foods). Data were collected from March to June 2020. The sampling frame for this study was publicly available information on the websites of selected preschools and primary schools, which included formal policy documents and any informal content available on the websites—including newsletters, enrolment packs, canteen menus, and information about programs that included educational and activity-based approaches that are part of the school curriculum or extra-curricular agenda.

### School selection

In South Australia, children can study in a government school (also referred to as public schools) or private schools (which are non-government schools comprising Independent and Catholic schools) [34]. For this study, data were extracted from publicly available websites of public preschools (kindergarten; children aged approximately 3–5 years) and primary schools (elementary level; children aged approximately 5–12 years) in the Greater Adelaide region [35] of South Australia. At the time of writing, primary school includes Reception to Grade 7. Only government preschools and primary schools were included in this study, as it is considered mandatory for government schools to adhere to the local-level policies and guidelines and encouraged but voluntary for Catholic or Independent private schools [36].

According to the Australian Bureau of Statistics [37], there are 219 preschools and 234 primary schools in the Greater Adelaide region of South Australia, which includes the Metro and Hills. Purposive sampling was adopted to ensure we captured a variety of schools in our sample that had information-rich websites related to the phenomenon of interest. In particular, since an important predictor of childhood overweight and obesity is socioeconomic status (SES) [38], schools of varying SES were sampled for demographic and socioeconomic diversity. Geographical location data in the form of postcodes of the schools (pre/primary) were used to determine the Index of Relative Socioeconomic Advantage and Disadvantage (IRSAD) score, which gave information about the broader socioeconomic context of the schools. The SES of the school was derived from the Socioeconomic Index for Australia sourced from the Australian Bureau of Statistics [37]. Postcode data were accessed from the Australian Urban Research Infrastructure Network, and the respective schools in various postcodes were assigned the IRSAD score [39, 40]. Using the IRSAD deciles, schools were categorized into low (IRSAD score 743–889), medium (IRSAD score 942–1005) and high SES (IRSAD score 1009–1127) groups.

### Procedure

All pages and sections of the schools’ websites were viewed and searched for all formal policies and any informal content around healthy eating, environmentally friendly aspects of food choice and the combination of both. Screenshots of relevant content from each preschool or primary school were taken and stored. This snapshot approach prevails over the variable nature of websites by taking a ‘static slice of a dynamic medium’ and examining that ‘slice’ at a certain point in time [41]. Data coding and analysis commenced while data were being collected, enabling us to determine when data saturation had been achieved and no new findings were emerging [42, 43].

### Data analysis

A content analysis was then undertaken whereby websites were reviewed and coded based on pre-determined categories of interest [44]: (i) healthy eating, (ii) environmentally friendly practices and (iii) low-waste healthy foods. Pre-determined categories initially guided the study, but themes and subthemes were identified and reanalysed throughout study progression. The researcher analysed the content for each category represented and not the extent of the representation. Our approach took a critical realist epistemology and followed the ‘ethnographic content analysis’ used by Altheide [41, 42] drawing in a recursive and reflexive movement between concept development-sampling-data, collection-data, coding-data and analysis interpretation. Analysis of content within each pre-determined category gave rise to three interrelated and repeated patterns across the school website content: ‘Policy’, ‘Promotion’ and ‘Implementation’.

In this study, Human Research Ethics Approval was not required as school websites are in the public domain. Furthermore, no identifying information is included in the data presented in this paper.

## Results

### Characteristics of the sample

The websites from a total of 18 preschools and primary schools were included in the analysis of this study. There were nine preschools and nine primary schools; with an equal number of each school type (*n* = 3) in each of the SES groups.

Descriptive statistics relating to the website content and included below were calculated for formal policies, excluding allergen management policies (since that was outside the scope of this study) and other content on implementation or promotional activities relating to nutrition, environment and the overlap between the two. Table I shows the percentage of the presence of nutrition- and environment-related policies within the sampled dataset of schools. Overall, 44% had publicly available nutrition policies, including 89% of the preschools and none of the primary schools. Most (83%) did not have an environment-related policy in place; the only policies observed were in preschools (*n* = 3, 33.3%). Table I also shows the presence of nutrition- and environment-related policies in preschools and primary schools, respectively, with SES segregation. There were no formal policies encompassing low-waste healthy foods in either preschools or primary schools.

**Table I.** Overall presence of nutrition and environment-related policies by type of school (N = 18)

	Policy	
Type of school	Nutrition	Environment
Preschool (*n* = 9)	8 (88.9%)	3 (33.3%)
Low (*n* = 3)	2 (66.7%)	0 (0%)
Medium (*n* = 3)	3 (100%)	1 (33.0%)
High (*n* = 3)	3 (100%)	2 (66.7%)
Primary school (*n* = 9)	0 (0%)	0 (0%)

Table II shows the percentage of preschool and primary school websites that contained promotional or implementation content on nutrition- and/or environment-related activities, other than the policy content. Overall, 88.9% had nutrition-related content, while 100% had environment-related content, and in 27.8% there was an overlap of nutrition and environment. While primary schools did not have formal policies (Table I) on either nutrition or environment, the majority of their websites depicted the promotion and implementation of healthy eating activities (77.8%) via curriculum, newsletters or websites, and a health advocacy group. All primary school websites (100%) contained environmental content, with promotion and implementation seen through educational practices, school values and philosophy, newsletter correspondence and community gardens.

**Table II.** Overall presence of at least one nutrition and environment-related promotional activities and implementation content by type of school (N = 18)

	Promotion and implementation		
Type of school	Nutrition	Environment	Overlap
Preschool (*n* = 9)	9 (100%)	9 (100%)	3 (33.3%)
Low (*n* = 3)	3 (100%)	3 (100%)	2 (66.7%)
Medium (*n* = 3)	3 (100%)	3 (100%)	1 (33.3%)
High (*n* = 3)	3 (100%)	3 (100%)	1 (33.3%)
Primary school (*n* = 9)	7 (77.8%)	9 (100%)	2 (22.2%)
Low (*n* = 3)	2 (66.7%)	3 (100%)	0 (0%)
Medium (*n* = 3)	3 (100%)	3 (100%)	1 (33.3%)
High (*n* = 3)	2 (66.7%)	3 (100%)	1 (33.3%)

### Thematic findings

The analysis presented here is structured according to the pre-determined categories of interest, and the patterns identified within and across them (policy; promotion; implementation).

### Healthy eating

#### Policy

It was common for content to refer explicitly to overarching policies and guidelines (such as the Right Bite strategy, National Dietary Guidelines or the Department for Education healthy eating guidelines) and to use them as rationale for justifying advice to parents. Preschools had different names for their food and health policies, such as ‘Healthy Food Policy’, ‘Healthy Food Guideline’, ‘Healthy Eating Policy’ and ‘Healthy Food Supply and Nutrition Policy’. Policies were present to inform parents that children were expected to bring a drink bottle containing water, two healthy snacks (such as two pieces of fruit or vegetable) and a healthy packed lunch that included *‘*a balanced intake of nutritious foods from the five recommended food groups’. Schools referred to processed energy-dense, nutrient-poor food items that were considered not healthy as *‘*junk food’ or ‘unsuitable foods’; hence children were asked not to bring such foods to schools within policy documents. One kindergarten informed parents via the newsletter—*‘*As per our Healthy Food Policy, unsuitable foods such as cakes, chips, chocolates and sweet biscuits will be returned to the child’s bag to be eaten at home’. This insinuated that the school environment should be devoid of ‘unhealthy’ foods.

**Fig. 1.**
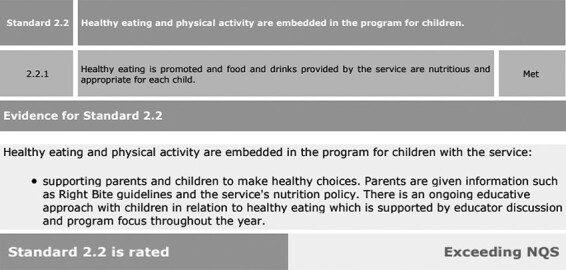
A preschool’s National Quality Standard Report on healthy eating.

Certain healthy eating policies comprehensively mentioned ‘food items containing preservatives and artificial colourings should be limited as much as possible’. Some policies included the ‘traffic light’ categories of the Right Bite strategy with examples of foods, to recommend more food items from the Green category, only one item from the Amber category, and no foods or have occasionally from the Red category. These guidelines also helped set limits on ‘special events’ food to twice a term to avoid or limit the consumption of unhealthy foods during celebrations in preschools. Moreover, much like the limits on special foods, fundraising activities involving food were expected to follow the healthy eating guidelines. One preschool displayed their National Quality Standard report on their website whereby they exceeded standards in the domain of healthy eating for children and provided parents with information about the Right Bite guidelines (see Fig. 1).

#### Promotion

Preschools and primary schools demonstrated their commitment to healthy eating through different promotion strategies. Healthy food guidance or lunchbox expectations were communicated to parents or guardians through various mediums such as the school website, newsletters, enrolment packs and school communication applications, as the responsibility to provide healthy snacks and lunches as per guidelines falls on them. For instance, when fewer fruits were being brought by children in one preschool, this information was conveyed via the newsletter to improve fruit consumption in schools.

There was the presence of healthy eating curriculum in some schools with examples including the ‘Eat a Rainbow’ lesson plan (eating fruits and vegetables of different colours), lunchbox investigations to promote healthy eating or the presence of a lunch care supervisor to teach children about food and nutrition. Health-oriented activities were also available for children such as preparing and cooking healthy foods in class. There was also an instance whereby one school supported the formation of a health and nutrition advocacy group that was led by parents, students and teachers, which helped address concerns of ‘junk food’ coming into school or acknowledging good nutritional choices. Parents or guardians were also welcomed to join the Governing Council in schools to be involved in schools’ activities and subsequently children’s health and well-being development.

#### Implementation

**Fig. 2.**
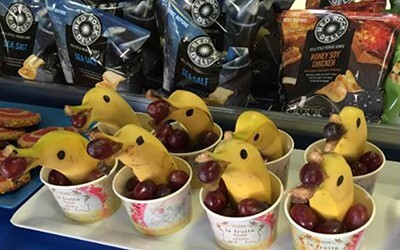
A school canteen running in line with the Right Bite Healthy Eating Guidelines—what is on offer includes fruit bowls (banana and grapes), potato chips (back) and sweet biscuits (left).

Despite the general representation of schools to support healthy eating, there were instances where practices deviated from policy. For example, in some schools, celebrations and activities were linked to unhealthy foods, as well as unhealthy canteen specials. Certain primary schools had unhealthy food items in their canteen menu, despite aiming to provide healthy and nutritious foods in line with the overarching policies. As a result, often both healthy and non-healthy choices were found in canteens, as shown in Fig. 2. A selection of canteen menus were colour coded in green, amber and red, in line with the Right Bite strategy to reflect the healthy and non-healthy choices available. Some school canteens claimed that they offer ‘healthy choices that are delicious and affordable’, and ‘aimed’ to provide nutritious food and serve healthy foods as specials; these schools clearly identified their healthy food and snacks options in addition to the regular items and promoted this via canteen menus or newsletters. The influence of policy on canteen foods was also seen as one primary school mentioned ‘Reduced or low fat products are used throughout the menu where possible’.

### Environmentally friendly practices

#### Policy

Three preschools had formal policies encompassing their respective environmentally friendly practices. One preschool had a ‘Sustainable Procedure’ and another had an ‘Environment Management Plan’. The third preschool’s website showed the ‘Wipe Out Waste’ program and policy through engagement with KESAB (Keep South Australia Beautiful) ‘environmental solutions’, a not-for-profit organization delivering environmental sustainability programs to schools. The content of these policies included schools’ vision and/or values including an environmental agenda, schools’ efforts to promote environmental awareness and education. Focus on environmentally friendly practices comprised reducing waste and packaging, the ‘Nude Food’ philosophy, recycling, gardening, composting and conservation of energy/resources.

#### Promotion

Preschools and primary schools that were sustainability oriented had values that included consideration for the environment, such as ‘Responsible towards nature’, ‘Environmentally aware’, ‘Sustainability’ and ‘Respectful interactions with our environments’. School philosophies which considered that the environment were in the form of slogans such as ‘better future for the planet’ and ‘providing an environment to understand sustainable practices’. School values also shaped the outcomes shared in school reports including current and future environment-related actions that were part of the schools’ agenda (such as the National Quality Standard report), which was publicly available in one preschool website. Schools often relayed information about sustainability and environmental activities via newsletters. Parental involvement in the schools’ sustainable actions was also welcomed.

#### Implementation

Various environment-oriented programs and activities within the school setting were found on websites of preschools and primary schools. In some instances, there were lunch waste and bin audits for waste minimization, encouraging ‘Nude Food’ through the use of washable containers, promoting less waste to landfill by minimizing or reducing packaging, and switching to environmentally friendly cutlery within the canteen. Moreover, some sustainable activities were carried out in collaboration with other members of the school and society. Other school activities that promoted environmental awareness included emphasis on recycling and upcycling through education, teaching waste and bin management, taking the children to the beach for cleaning or promoting the use of sustainable library bags. The presence of a community garden in schools to grow fruits and vegetables was also common in some preschools and primary schools, as emphasis was laid on gardening, composting and food scrap handling. Furthermore, the inclusion of environmental awareness events into the school’s calendar such as ‘Earth Hour’ or ‘Earth Day’ was captured within this domain.

### Low-waste healthy foods

#### Policy

We found no formal policies encapsulating the combination of healthy eating and environmentally friendly practices. However, ‘Nude Food’ information was briefly mentioned in the healthy food policies/guidelines of three preschools and one preschool’s sustainability procedure. There was also an occurrence where parents were asked to be mindful about portion sizes to minimize uneaten foods being wasted, and this information was conveyed in the ‘Healthy Food Guideline’ of a preschool.

#### Promotion

**Fig. 3.**
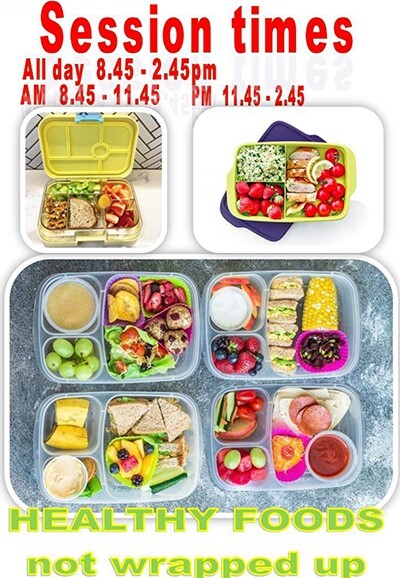
Example of ‘healthy foods not wrapped up’ information relayed via a school newsletter.

Various examples of encouragement of healthy foods and less landfill waste were found in the informal content analysed. Although not driven by a formal policy, there was emphasis on healthy foods and ‘naked foods’ that were not wrapped up, and these expectations were conveyed on websites, Parent Information Booklet and newsletters (see Fig. 3). This was also integrated into the curriculum in one preschool. Tips on healthy and waste-free lunches were shared on some websites, and certain preschools implemented KESAB’s ‘Wipe Out Waste’ program.

#### Implementation

**Fig. 4.**
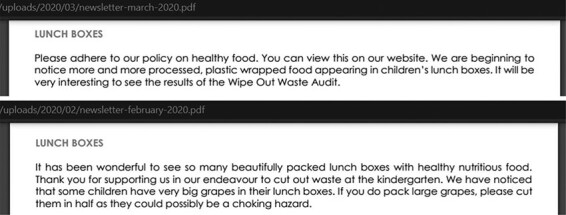
Newsletter update across 2 months regarding lunchbox contents in a preschool.

Implementation within the overlapping domains of healthy eating and environmentally friendly practices was not particularly apparent from the content analysis process. However, there was an instance where one preschool’s newsletter informed parents about healthy and waste-free lunchboxes found at the start of term, the rate of which declined the following month with more processed and plastic wrapped foods observed in lunchboxes (see Fig. 4).

## Discussion

This study explored policies and programs around healthy eating, environmentally friendly practices and low-waste healthy foods, among a sample of preschools and primary schools across different socioeconomic areas, as evidenced by their public facing websites. We found that healthy eating policies and programs were more common compared to those encouraging environmentally friendly practices; the combination of both aspects were lacking within documented policies and programs. Our interest in examining school websites for the prevalence of policies and programs stemmed from the view that this digital platform is used by respective institutions to provide relevant information regarding the operations of the pre/primary school. Advocacy efforts and information are often portrayed on the websites for the parents or guardians of students both prospective and currently enrolled [45].

Preschools and primary schools are important settings for the development of healthy eating and eco-friendly habits. The involvement of various stakeholders including children, parents, teachers and the school community for childhood development and well-being is vital for the long-term success of policies and programs created in the school environment context. Some preschools also acknowledged their role to support families to ensure optimal health and nutritional outcomes of their children. There was a clear acknowledgement of the roles and responsibilities of parents or guardians in providing their children with a packed lunchbox. The presence of healthy eating policies and guidelines tends to shape parental responsibilities in the provision of snacks and lunches for their children. Hence, lunchbox expectations were also often communicated to parents or guardians through various mediums such as the school website, newsletters, enrolment packs and school communication applications. The mention of unhealthy food items being sent back home, notably in the preschool context, took the role of guidance and policy further by refusing to allow the consumption of products that were not aligned with policy. Preschools had policies in place, and there was evidence of these policies being enforced, whereas primary schools did not. Additionally, parental involvement encouraged through the Governing Council to be involved in the review of the healthy eating policies/guidelines and other school matters further underlines their key role in influencing what children bring in their lunchbox. This parental or familial role to influence children’s food consumption behaviour has been well established in the literature [46, 47]. Hence, engagement with parents and their support towards the school’s healthy food and sustainability policies and programs are crucial.

Canteens play an integral role in providing food, as well as educating and modelling a healthy food environment [48]. The Right Bite strategy guides the food and drink supply in canteens, to ensure that healthy choices are available in South Australian schools and preschools [28]. While a mix of healthy and unhealthy foods items were found in canteen menus, it is worth noting that the biggest effect of the Right Bite guidelines was visible in the design of lunch menus where green, amber and red foods were clearly identified based on colour coded text and is also a positive educational tool for students and families. Moreover, the healthy eating guidelines and policies found in preschools were elaborate and in line with the national and local guidelines, which involves grouping foods into the categories of the ‘traffic light’ system [49] (green, amber and red), to convey the expectation of more green (healthy and minimally processed) foods to be included in lunchboxes. Hence, this ‘traffic light’ guidance can be useful beyond the canteen setting, although lunchboxes coming from home is currently not covered by the aforementioned guidance [12].

Although only three preschools had a formal sustainability procedure or policy in place, the majority of the preschools and primary schools that were devoid of policies and/or guides had various programs and activities that promoted environmental awareness and sustainable practices. Children were supported and enabled to carry out environmentally friendly practices. The promotion of the ‘Nude Food’ program for healthy eating and reduction of landfill waste in some schools also opened the avenue of providing education around waste management and conducting routine waste audits to minimize lunchbox and packaging waste. Some schools’ engagement with a local environmental agency and encouragement to families to provide nutritious and waste-free lunchboxes suggested that there is scope to develop stakeholder engagement to improve outcomes in the context of environmentally friendly practices. This suggests that there is a basis on which policy could be developed in primary schools in particular to support schools to achieve improved nutritional and environmental outcomes.

The challenges of packing and consuming a nutritious and low-waste lunchbox are an underrated public and planetary health concern. The responsibility of healthy eating behaviour development and encouraging environmentally friendly actions was found to be diffused across multiple stakeholders (i.e. schools, environmental agencies, policymakers, education department, school staff members, parents and schoolchildren). Where the responsibility of the intersection of healthy eating and environmentally conscious practices lies is unclear and is possibly an idea worth future consideration. Finally, although the nexus of the intersection of healthy eating and environmentally friendly practices was underdeveloped, it could certainly be mobilized to increase the prevalence of low-waste healthy foods within the school setting. Thus, there seems to be scope for the development of formal policies that merge the nutritional and sustainable aspects for better health and environmental outcomes through schoolchildren’s eating behaviour.

### Limitations

In considering the analysis presented here, it should be noted that the desktop review of published materials on school websites does not capture other aspects of the school environment, unpublished policies/guidelines or programs and the results of policy implementation. Moreover, we have only reviewed websites of schools in the Greater Adelaide region not including schools in the regional areas. Therefore, generalizability of findings is a limitation worth noting in a geographical context, and the school environment context where the food consumption models may vary. Nevertheless, the sample of schools included in this study represents the majority of the population residing in South Australia [50].

### Conclusions

In this study, preschools had a strong presence of healthy eating policies, limited environment/sustainability policies and no policies around the overlap of both aspects. Formal policies around healthy eating, environmentally friendly practices and the combination of both were not found on the websites of primary schools. Initiatives or activities through school programs around healthy eating were guided by policies and/or guidelines. Government and local-level authorities serve as a reference point for schools to base their decisions on how to structure policies. However, environmentally friendly practices were mentioned as activities that were being promoted or encouraged and therefore seemed to be more ad hoc in nature. Similarly, expectations of bringing healthy and eco-friendly foods were mentioned, but they were not part of formal policy documents. Hence, clearer and well-defined policies are warranted, especially those that suit the Australian packed lunchbox model managed by parents and particularly those that involve the overlap of nutrition and sustainability. We therefore call for actions to re-think food consumption in school, incorporating an environmental agenda onto the well-established nutrition policies and guidelines.

### Implications for health and sustainability

This study provided an examination of the context of the existing school food environment in South Australian preschools and primary schools, and what aspects were considered important and subsequently shared on public domains. Given that there were no policies in place around the ‘co-benefits’ of healthy eating and environmentally friendly actions, but plenty of encouragement by the schools, this is an avenue worth developing within the school environment. Furthermore, this study can inform future research that could analyse ‘lived experiences’, such as auditing children’s consumptions patterns within the school setting. These outcomes combined will help shape the scope of new policies and programs that could merge the nutrition and environment lens together as part of an inter-sectoral approach to improve children’s dietary habits and sustainability outcomes.
